# Angiotensin-(1-7) and Central Control of Cardiometabolic Outcomes: Implications for Obesity Hypertension

**DOI:** 10.3390/ijms252413320

**Published:** 2024-12-12

**Authors:** Victoria L. Vernail, Lillia Lucas, Amanda J. Miller, Amy C. Arnold

**Affiliations:** 1 Department of Neural and Behavioral Sciences, Pennsylvania State University College of Medicine, Hershey, PA 17033, USA; vvernail@pennstatehealth.psu.edu (V.L.V.); or ajmiller@lvc.edu (A.J.M.); 2 Department of Physical Therapy, Lebanon Valley College, Annville, PA 17003, USA

**Keywords:** renin–angiotensin system, blood pressure, metabolism, obesity, brain

## Abstract

Hypertension is a leading independent risk factor for the development of cardiovascular disease, the leading cause of death globally. Importantly, the prevalence of hypertension is positively correlated with obesity, with obesity-related hypertension being difficult to treat due to a lack of current guidelines in this population as well as limited efficacy and adverse off-target effects of currently available antihypertensive therapeutics. This highlights the need to better understand the mechanisms linking hypertension with obesity to develop optimal therapeutic approaches. In this regard, the renin–angiotensin system, which is dysregulated in both hypertension and obesity, is a prime therapeutic target. While research and therapies have typically focused on the deleterious angiotensin II axis of the renin–angiotensin system, emerging evidence shows that targeting the protective angiotensin-(1-7) axis also improves cardiovascular and metabolic functions in animal models of obesity hypertension. While the precise mechanisms involved remain under investigation, in addition to peripheral actions, evidence exists to support a role for the central nervous system in the beneficial cardiometabolic effects of angiotensin-(1-7). This review will highlight emerging translational studies exploring the cardiovascular and metabolic regulatory actions of angiotensin-(1-7), with an emphasis on its central actions in brain regions including the brainstem and hypothalamus. An improved understanding of the central mechanisms engaged by angiotensin-(1-7) to regulate cardiovascular and metabolic functions may provide insight into the potential of targeting this hormone as a novel therapeutic approach for obesity-related hypertension.

## 1. Introduction

Cardiovascular disease (CVD) is not a disease unique to modern history, with ancient Egyptian, Chinese, and Greek historical texts referencing “hard pulse disease” and blood disorders [1,2,3]. It was not until the 20th century, when CVD became the leading cause of death in the United States [4], that the focus of research shifted towards more modern preventative measures, diagnostic tools, and treatment [5]. Despite major advancements in research and therapeutic approaches, CVD remains the leading cause of death globally. The risk factors for developing CVD range from poor diet and sedentary behavior to certain medical conditions, with the greatest independent predictor being hypertension [6]. The prevalence of hypertension in adults is approximately 46%, with this prevalence increasing with age [7]. Of particular importance, there is a strong correlation between rates of hypertension and obesity, with an estimated 70% of hypertension cases attributed to obesity [8,9]. Despite this clinical correlation, the molecular mechanisms connecting obesity with hypertension remain poorly understood. This is illustrated by the finding that currently available therapeutics often have limited efficacy and off-target effects. For example, some classes of antihypertensive drugs, such as β-blockers and diuretics, are associated with poor metabolic outcomes such as weight gain, hyperglycemia, and insulin resistance [10]. Conversely, several anti-obesity therapeutics have been withdrawn from the market due to adverse cardiovascular effects, including elevated blood pressure [11,12]. Therefore, it is important to gain a greater understanding of the etiology of hypertension, and its connection with obesity, to identify new effective therapeutic targets with a positive cardiometabolic profile.

The renin–angiotensin system (RAS) is well recognized to be dysregulated in both hypertension and obesity and to contribute to the development and pathogenesis of these conditions. Many first-line antihypertensive medications are currently available to block the vasoconstrictor arm of the RAS, including direct renin inhibitors, angiotensin-converting enzyme inhibitors (ACEIs), angiotensin II type 1 receptor blockers (ARBs), and mineralocorticoid antagonists [13]. Despite having a positive cardiometabolic profile, these therapies are not able to effectively control blood pressure in many patients and are plagued by side effects due to off-target actions on other signaling pathways. More recent evidence suggests that the targeting of the vasodilatory arm of the RAS, characterized by the protective hormone angiotensin (Ang)-(1-7), may provide an improved therapeutic approach over currently available RAS inhibitors. In support of this, recent studies have shown that Ang-(1-7) has a positive cardiometabolic profile in hypertensive, obese, and diabetic rodent models, including blood pressure lowering and improvements in glucose homeostasis, energy balance, and lipid metabolism [14,15]. While numerous mechanisms have been proposed, the protective cardiometabolic effects of Ang-(1-7) appear to involve, at least in part, a central mechanism of action. This review will highlight emerging translational studies exploring the cardiovascular and metabolic regulatory actions of Ang-(1-7) [16,17], with an emphasis on its central actions in brain regions including the brainstem and hypothalamus. An improved understanding of the central mechanisms engaged by Ang-(1-7) to regulate integrated cardiometabolic function may inform the potential of targeting this hormone for the treatment of obesity hypertension and related cardiometabolic disorders.

## 2. Angiotensin II Pathways

**Figure 1:**
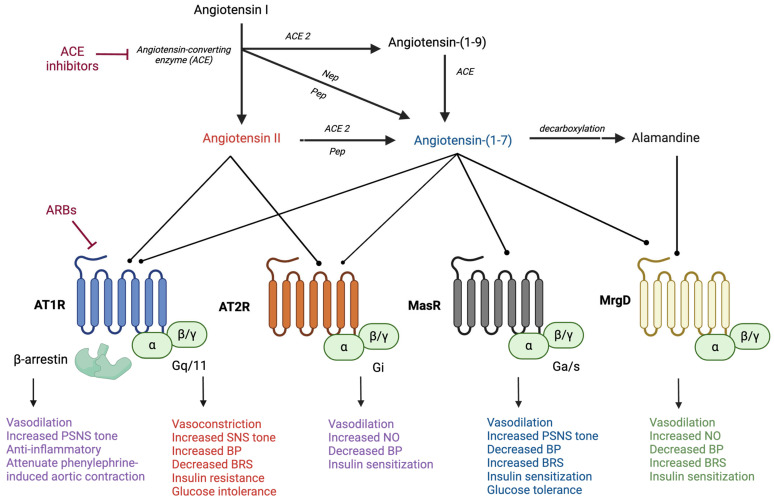
RAS hormones elicit differential cardiometabolic effects by binding to specific angiotensin receptors. AT1R: angiotensin II type 1 receptor; AT2R: angiotensin II type 2 receptor; MasR: angiotensin-(1-7) Mas receptor; MrgD: Mas-related G protein-coupled receptor; SNS: sympathetic nervous system; PSNS: parasympathetic nervous system; BP: blood pressure; BRS: baroreflex sensitivity; NO: nitric oxide. Created in BioRender. Arnold, A. (2024) https://BioRender.com/e19f735 (accessed on 11 December 2024).

The RAS is well recognized for its participation in the regulation of blood pressure and fluid and electrolyte balance, with more recently identified roles in other physiological functions, including energy balance and glucose homeostasis. In the classical vasoconstrictor arm of the RAS, the precursor angiotensinogen is cleaved by the enzyme renin to form Ang I, which is then cleaved by ACE to form Ang II (Figure 1). Elevated circulating levels of Ang II are often observed in both hypertension and obesity in animal models and clinical populations. Ang II binds to type 1 G protein-coupled receptors (AT1Rs) to promote cardiometabolic complications, including elevations in blood pressure and impairments in insulin sensitivity and glucose tolerance [15,18]. Numerous peripheral and central mechanisms have been associated with the deleterious cardiometabolic effects of Ang II, including vasoconstriction, aldosterone release, sympathetic activation, impaired arterial baroreflex sensitivity, and the activation of pro-inflammatory and immune pathways. Ang II can also bind to its type 2 G protein-coupled receptors (AT2Rs) to induce effects opposite to AT1R stimulation, such as vasodilation, although these receptors have more limited affinity and tissue expression. In addition to G protein-dependent intracellular signaling, the activation of AT1Rs by Ang II can engage intracellular β-arrestin pathways to promote vasodilation and cardioprotection. Research is ongoing to test the ability of AT1R β-arrestin-biased agonists and AT2R agonists to engage the protective effects of Ang II in cardiovascular-related diseases. The cardiometabolic regulatory actions of Ang II and the related signaling pathways and tissue-specific mechanisms will not be a focus of this review, as they have been previously summarized extensively [18,19,20,21].

Drugs blocking Ang II activity, such as ACEIs and ARBs, are first-line recommendations for hypertension treatment. That being said, current hypertension guidelines do not provide specific recommendations for obesity hypertension, with few clinical trials examining antihypertensive therapy’s safety and efficacy in this population [22]. ACEIs and ARBs have a positive cardiometabolic profile and are known to lower blood pressure, improve insulin sensitivity, and reduce the risk of new-onset diabetes, making them attractive options for obesity hypertension [23]. Despite this, these therapies are often not sufficient to control blood pressure on their own [24]. In addition, ACEIs are not tolerated in a significant number of patients due to a dry cough as a result of bradykinin accumulation and subsequent bronchoconstriction, and more rarely, they can produce angioedema [25,26]. Given these side effects and that less than half of those diagnosed with hypertension have their blood pressure successfully controlled by these classes of drugs [27], there is a growing need to investigate additional antihypertensive targets, particularly in the context of obesity. Of interest, both ACEIs and ARBs increase the levels of endogenous Ang-(1-7), which has been shown to contribute to the beneficial cardiometabolic effects of these therapies in obese and hypertensive rodents [28,29]. Thus, the direct targeting of Ang-(1-7) may provide an alternate approach to improve the cardiometabolic function in obesity hypertension while avoiding the off-target side effects seen with current RAS-blocking therapies.

## 3. Angiotensin-(1-7) Pathways

Ang-(1-7) is a heptapeptide that is formed largely from the cleavage of Ang II by angiotensin-converting enzyme 2 (ACE2). Ang-(1-7) can also be formed to a lesser extent from the cleavage of Ang I by endopeptidases, such as neprilysin, or from Ang-(1-9) by ACE (Figure 1) [30]. Ang-(1-7) is metabolized via decarboxylation to alamandine, or to additional peptides such as angiotensin-(2-7) or angiotensin-(1-5) via ACE or endopeptidases. Initially, Ang-(1-7) was described as a byproduct of the RAS and was not thought to be biologically active. Interest in Ang-(1-7) as a biologically active peptide, however, grew with the discovery of the Mas receptor (MasR) in 2003. Since then, numerous studies have shown that the activation of Ang-(1-7) pathways opposes the deleterious Ang II axis to improve physiological outcomes in cardiovascular disease and numerous other disease states. Relevant to this review, accumulating evidence shows that systemic Ang-(1-7) treatment improves both cardiovascular and metabolic outcomes in rodent models of cardiovascular-related diseases such as hypertension, diabetes, obesity, heart failure, atherosclerosis, stroke, and myocardial infarction [31,32,33,34,35,36].

### 3.1. Angiotensin-(1-7) Mas Receptors

Ang-(1-7) binds with high affinity to MasR, a G protein-coupled receptor. MasR is found throughout the periphery, including in cardiometabolic regulatory organs such as the heart, kidney, blood vessels, skeletal muscle, and adipose tissue [37,38]. The Ang-(1-7) stimulation of MasR results in enhanced nitric oxide (NO) release and reduced oxidative stress to promote vasodilation [39]. The effects of Ang-(1-7) on NO and oxidative stress are MasR-dependent, as they are blocked by the selective MasR antagonist A779 [40,41]. Ang-(1-7)-mediated NO release is thought to be related to the activation of the PI3K/AKT pathway [16]. Ang-(1-7) can also inhibit ERK1/2 phosphorylation to reduce cell proliferation [36] and prevent cardiac remodeling [42]. In addition to Ang-(1-7), synthetic MasR agonists, including AVE 0991 and CGEN-8856S, induce vasodilatory effects in animal studies [43]. While the majority of the in vivo physiological actions of Ang-(1-7) appear MasR-mediated, as they are prevented by either A779 or the genetic MasR deletion, a few recent reports have suggested either no effect of Ang-(1-7) on MasR or that MasR interacts with other receptors, including AT1R, AT2R, bradykinin B2, and endothelin B receptors [44]. For example, a few studies have reported that Ang-(1-7) acts as a biased agonist of AT1R, binding through the β-arrestin pathway to reduce cardiac hypertrophy and phenylephrine-induced aorta contraction [45,46]. While some studies have shown that AT2R and bradykinin B2 receptor antagonists can prevent Ang-(1-7)-mediated vasodilatory responses, other studies have shown no role for these receptors in Ang-(1-7) effects [47,48,49]. Ang-(1-7) has also been shown to bind with less potency to Mas-related G protein-coupled receptors (MrgDs) to increase cAMP in cultured cells [50]; however, whether this occurs in vivo to elicit cardiovascular effects is unknown. Overall, research is ongoing to clarify the relationship between Ang-(1-7) and MasR and the potential interactions of this hormone with other receptors, particularly in specific tissue and cell types.

### 3.2. Cardiometabolic Effects of Systemic Angiotensin-(1-7) Administration

The chronic systemic treatment with Ang-(1-7) is well-established to lower blood pressure in animal models of hypertension [33,51]. This depressor effect has been associated with peripheral and central mechanisms of action, including enhanced NO release, vasodilation, anti-inflammatory and anti-oxidant effects, sympathetic nervous system inhibition, and parasympathetic nervous system facilitation [13]. While the information is more limited, alamandine appears to produce similar effects as Ang-(1-7) in animal models, including reductions in blood pressure and cardiac and renal damage in hypertensive rats, but via binding MrgDs [52,53]. The cardioprotective effects elicited by the activation of MrgDs are reported to involve the activation of NO signaling pathways [54].

While the cardiovascular actions of Ang-(1-7) have been well studied, the impact of this hormone on metabolic outcomes has only recently been explored. In addition to positive cardiovascular effects, systemic Ang-(1-7) infusion improves metabolic outcomes in animal models of hypertension, obesity, diabetes, cardiometabolic syndrome, and aging. This includes improvements in insulin sensitivity and secretion, glucose tolerance, lipid profile, and energy balance [14,15]. In terms of insulin sensitivity, Ang-(1-7) activates intracellular insulin signaling and promotes glucose transport activity in insulin-sensitive tissues in a MasR-dependent manner [14,55]. Furthermore, recent studies have demonstrated that MrgDs play a role in metabolic regulation. Enhanced MrgD expression in the liver may contribute to the insulin-sensitizing effects of the systemic administration of the Ang-(1-7) analog A-1317 in rats [56], and global MrgD knockout mice have reduced thermogenic brown adipose tissue [57]. Taken together, the vasodilatory arm of the RAS may be facilitated by actions at both MasR and MrgD. While not a focus of this review, systemic Ang-(1-7) infusion is also protective in numerous other conditions, including stroke, traumatic brain injury, cognitive disorders, and gastrointestinal disease [58,59,60].

## 4. Central Angiotensin-(1-7) and MasR

In addition to the peripheral sites of action, accumulating evidence supports that RAS hormones engage the central nervous system to regulate cardiovascular and metabolic functions. Under normal conditions, circulating Ang peptides do not cross the blood–brain barrier; however, they can access the central nervous system through circumventricular organs such as the median eminence, subfornical organ, organum vasculosum of the lamina terminalis, and area postrema [61]. These circumventricular organs, which lack a functional blood–brain barrier, contain AT1R and MasR and project information to cardioregulatory brain regions, including the hypothalamus and brainstem [62]. In addition, in hypertension and obesity, the structure and function of the blood–brain barrier are disrupted and more permeable [63,64,65,66,67], which could permit easier access of Ang peptides to cardioregulatory areas controlling blood pressure and metabolic function [68]. Ang peptides are also reported to be synthesized throughout the brain via a local RAS, although the level of independence of these local systems from the circulation remains controversial [69,70,71]. AT1R and MasR are found throughout autonomic nervous system pathways to influence sympathetic and parasympathetic neurotransmission, including on preganglionic neurons, ganglia, nerve terminals, and neuron cell bodies in cardiometabolic regulatory brain regions. Immunoreactivity for Ang II and Ang-(1-7) has also been reported throughout the brain, including in cardiometabolic regulatory regions in the brainstem and hypothalamus such as the nucleus tractus solitarius (NTS), caudal ventrolateral medulla (CVLM), rostral ventrolateral medulla (RVLM), arcuate nucleus (ARC), and paraventricular nucleus (PVN) [13]. These Ang peptides and receptors have also been reported in additional brain regions, such as the hippocampus, cortex, amygdala, and basal ganglia, to influence cognition, reward, and other physiological functions [72].

### Cardiometabolic Effects of Intracerebroventricular Angiotensin-(1-7) Administration

To determine the importance of the central nervous system to Ang-(1-7) effects, initial studies employed intracerebroventricular (icv) drug administration. In terms of cardiovascular function, one study showed that the icv Ang-(1-7) infusion increased the baroreflex control of the heart rate in conscious rats, while intravenous administration had no effect, supporting a centrally mediated mechanism for the Ang-(1-7) modulation of arterial baroreflex sensitivity [73]. Additional studies have shown that icv Ang-(1-7) infusion restores cardiac autonomic balance, lowers blood pressure, and decreases cardiac hypertrophy in rat models of hypertension [74,75,76]. One of these studies showed that improvements in blood pressure and cardiac hypertrophy with central Ang-(1-7) were prevented by A779, supporting a MasR-dependent mechanism [77]. The improved cardiovascular function in hypertensive rats following icv Ang-(1-7) is associated with enhanced NO release, reduced AT1R and ACE gene expression, anti-inflammatory responses, and decreased neuronal apoptosis in the brain [78,79]. These findings have been extended to other species, with icv Ang-(1-7) lowering blood pressure and restoring autonomic function in a sheep model of fetal programming [80]. Similar to Ang-(1-7), icv alamandine infusion improves baroreflex sensitivity in rats [81]. Emerging evidence suggests that icv Ang-(1-7) also improves metabolic outcomes. In fructose-fed rats, chronic icv Ang-(1-7) reduces glucose and insulin levels and improves glucose tolerance, in addition to lowering blood pressure and cardiac sympathetic tone [82]. A recent report also showed that the icv injection of Ang-(1-7) induces brown adipose thermogenesis in a MasR-dependent manner in Siberian hamsters [83]. Overall, central Ang-(1-7) infusion has been shown to improve cardiovascular and metabolic outcomes in animal models of hypertension, fetal programming, and cardiometabolic syndrome.

## 5. Ang-(1-7) Modulation of Cardiometabolic Function via the Brainstem and Hypothalamus

**Figure 2:**
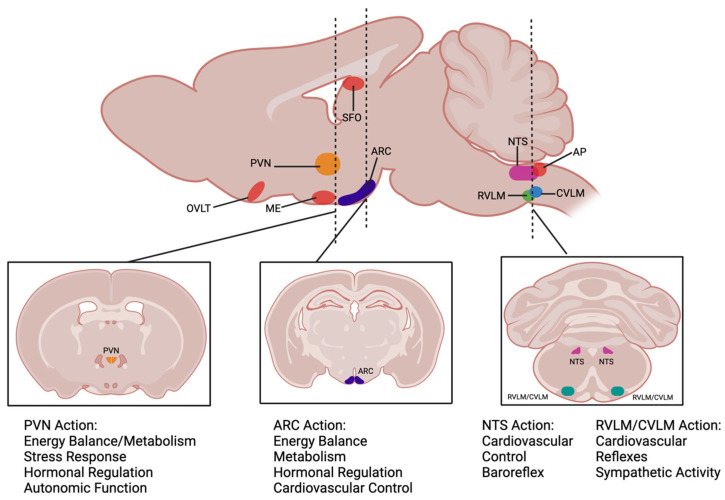
Cross sections of cardiometabolic nuclei in the hypothalamus and brainstem of the mouse brain and their major physiological functions. PVN: the paraventricular nucleus of the hypothalamus; ARC: the arcuate nucleus of the hypothalamus; NTS: the nucleus tractus solitarius; RVLM: the rostral ventrolateral medulla; CVLM: the caudal ventrolateral medulla. The areas highlighted in red represent the circumventricular organs. OVLT: the organum vasculosum of the lamina terminalis; ME: the median eminence; SFO: the subfornical organ; AP: the area postrema. Dotted lines represent the approximate location of the cross sections displayed. Created in BioRender. Arnold, A. (2024) https://BioRender.com/o23u712 (accessed on 13 November 2024).

Since icv administration does not provide information on specific brain regions, to further understand the central mechanism of action, Ang-(1-7) pathways have also been studied in the discrete brainstem and hypothalamic nuclei implicated in cardiovascular and metabolic control, such as the NTS, CVLM, RVLM, ARC, and PVN (Figure 2). Importantly, MasR immunofluorescence or gene expression has been detected in all of these brain regions in rodents [62,84]. As described below, several studies have examined the effects of acute Ang-(1-7) microinjection or MasR antagonism in these brainstem and hypothalamic regions on cardiovascular outcomes. There are, however, very few reports examining the impact of Ang-(1-7) on metabolic outcomes controlled by these regions.

### 5.1. Nucleus Tractus Solitarius

Blood pressure is, in part, regulated by the sympathetic and parasympathetic branches of the autonomic nervous system, which innervate cardiovascular end organs [85]. Changes in systemic blood pressure are detected by stretch-sensitive arterial baroreceptors in the aortic arch and carotid sinus. These baroreceptors are innervated by the vagus and glossopharyngeal nerves, which project to the NTS of the brainstem, to modulate sympathetic and parasympathetic neurotransmission [86,87]. Thus, the NTS is critical for the maintenance of cardiovascular stability via its ability to integrate input from arterial baroreceptors to appropriately modulate autonomic transmission to cardiovascular end organs as part of the arterial baroreceptor reflex. MasR immunofluorescence has been observed in the rostrocaudal region of the NTS in rats [62], the region receiving baroreceptor inputs. Consistent with this, the microinjection of Ang-(1-7) within the NTS improves the baroreflex function in both normotensive and hypertensive rats [13]. This is in opposition to the known effects of Ang II to suppress baroreflex function in the NTS. Despite opposing effects on baroreflex function, the microinjection of either Ang-(1-7) or Ang II into the NTS lowers blood pressure and heart rate in normotensive rats, with a biphasic depressor–pressor effect observed at high doses [88]. The NTS microinjection of Ang-(1-7) also reduces blood pressure and improves baroreflex sensitivity in hypertensive rats [89]. The cardiovascular effects of Ang-(1-7) in the NTS appear mediated by a MasR-dependent mechanism [90].

### 5.2. Caudal Ventrolateral Medulla

The stimulation of the NTS by baroreceptor afferent neurons causes glutamate release onto the CVLM [91], which is largely composed of sympathoinhibitory neurons and directly projects to the RVLM to restrain sympathetic outflow and lower blood pressure [92]. The unilateral microinjection of Ang-(1-7) into the CVLM decreases blood pressure in hypertensive and normotensive rats, with no effect on heart rate, similar to the effects of Ang II in this region [93]. While Ang II and Ang-(1-7) both lower blood pressure in the CVLM, they appear to act through different cellular mechanisms. The depressor effect of Ang II in the CVLM occurs through glutamatergic mechanisms, whereas enhanced GABAergic transmission contributes to Ang-(1-7) effects [94]. Enhanced NO activity may also contribute to the ability of Ang-(1-7) to lower blood pressure in the CVLM [40,95]. In support of this, the depressor effect of Ang-(1-7) in the CVLM is blocked by pretreatment with an NO synthase inhibitor, with no effect on Ang II responses [96]. Alamandine, a metabolite of Ang-(1-7), also lowers blood pressure when microinjected into the CVLM of normotensive, but not hypertensive, rats through an AT2R-mediated mechanism [97].

### 5.3. Rostral Ventrolateral Medulla

The RVLM is considered the major vasopressor center of the brainstem due to its control of sympathetic outflow to the periphery via monosynaptic connections to sympathetic preganglionic neurons [98]. RVLM stimulation increases cardiac and renal sympathetic activity to elevate heart rate and induce vasoconstriction and sodium and fluid retention. The RAS is well recognized to interact with RVLM circuits modulating cardiovascular sympathetic output [98]. For example, Ang II microinjection into the RVLM increases blood pressure via glutamate release in normotensive and hypertensive rats [99]. Interestingly, Ang-(1-7) and alamandine also increase blood pressure when injected into the RVLM of rats [100,101,102]. Conversely, the microinjection of either an ARB or MasR antagonist into the RVLM lowers blood pressure, indicating that endogenous Ang II and Ang-(1-7) contribute to tonic maintenance of blood pressure in this brainstem region. Both Ang II and Ang-(1-7) microinjected into the RVLM also elevate renal sympathetic nerve activity in rats [103]. The elevations in blood pressure and heart rate elicited by the Ang-(1-7) injection appear dose dependent and MasR-mediated [104]. It has been suggested that the pressor and sympathoexcitatory effects of Ang-(1-7) in the RVLM are due to the activation of MasR on astrocytes, resulting in the release of gliotransmitters, glutamate, and ATP [101]. The pressor actions elicited by Ang-(1-7) injection into the RVLM are contrary to the depressor effect seen when this hormone is administered systemically, intracerebroventricularly, or microinjected into other cardioregulatory brain regions. The overexpression of ACE2 in the RVLM, which should decrease Ang II and increase Ang-(1-7) levels, also lowers blood pressure in hypertensive rats, illustrating the complexity of this circuit [105].

### 5.4. Arcuate Nucleus

In addition to the brainstem, the hypothalamus controls both blood pressure and metabolic outcomes through interactions between the peripheral and central nervous systems [106]. The dysfunction of hypothalamic neural circuits has been implicated in hypertension, obesity, and metabolic syndromes [107]. In particular, the ARC is a vital hypothalamic region regulating both cardiovascular and metabolic functions, as it responds to circulating hormones (e.g., insulin, leptin, RAS peptides) to modulate downstream brain regions involved in the control of blood pressure and energy balance [108]. The ARC contains two primary neuronal subpopulations that have been implicated in cardiometabolic function: proopiomelanocortin (POMC) and agouti-related protein (AgRP). Within the ARC of mice, MasR gene expression is observed in glutamatergic and GABAergic POMC neurons as well as in GABAergic AgRP neurons [84]. Despite this, there have been no studies examining either the cardiovascular or metabolic effects of Ang-(1-7) administration in the ARC. Ang II AT1aR gene expression is observed in a subset of AgRP neurons expressing SSt3, with these receptors involved in the integrative control of the resting metabolic rate in transgenic mouse models [84,109]. One report showed that the Ang II microinjection into the ARC increases sympathetic activity in male and female rats via AT1R activation [110], but there have been no studies examining the metabolic effects of Ang II in this brain region. Since there is a differential expression of AT1R versus MasR within neuronal subtypes in the ARC, it is likely that anatomically distinct pathways and functions will be observed for Ang II versus Ang-(1-7) in this region; however, this remains to be explored.

### 5.5. Paraventricular Nucleus

The hypothalamic PVN, which receives afferent information from the ARC and other brain regions, is involved in the autonomic control of metabolism, stress, and cardiovascular activity [111]. Stimulation of the PVN is associated with sympathoexcitation and increased blood pressure [112]. MasR immunofluorescence has been reported in the PVN, including in the parvocellular and magnocellular subdivisions [62]. The Ang-(1-7) microinjection into the PVN increases blood pressure and the cardiac sympathetic afferent reflex (CSAR) in hypertensive and normotensive rats, similar to the effects of Ang II in this brain region [113]. Interestingly, the simultaneous injection of Ang-(1-7) and Ang II produces an additive effect, with a greater increase in the CSAR than either drug alone. An increase in the CSAR has been related to the development of chronic heart failure in rats and humans [114]. Microinjection of A779 into the PVN conversely reduces blood pressure and prevents CSAR activation in rats with chronic heart failure [114,115], suggesting that endogenous Ang-(1-7) contributes to tonic sympathetic and blood pressure regulation in this brain region. While the levels of ACE2 and Ang-(1-7) are not different between hypertensive and normotensive rats [116], the overexpression of ACE2 in the PVN attenuates the pressor responses seen with Ang II and Ang-(1-7) microinjection [117]. Similar to Ang-(1-7), the microinjection of alamandine into the PVN increases blood pressure and sympathetic activity in spontaneously hypertensive rats [118]. The pressor response following the microinjection of RAS hormones into the PVN may be due in part to the stimulation of downstream projections to regions including the RVLM to enhance sympathetic nerve activity and regulate blood pressure [111]. These robust signals may also be modulated by input from the ARC onto the PVN [106]. Ang-(1-7) injection into the PVN also induces vasopressin release, which appears mediated by AT1R and AT2R [30]. While several studies have shown that Ang-(1-7) modulates cardiovascular outcomes in the PVN [119], there are currently no reports of metabolic actions of this hormone in this hypothalamic region.

## 6. Clinical Implications

Based on findings from animal models, efforts are ongoing to determine the potential for translation of Ang-(1-7) cardiovascular and metabolic effects in clinical populations. In terms of cardiovascular outcomes, initial studies focused on the ability of acute Ang-(1-7) administration to promote vasodilation in isolated arterial beds. Ang-(1-7) has been shown to enhance NO release and dilate isolated arterioles in patients with coronary artery disease [120]. Acute intra-arterial Ang-(1-7) infusion also dilates brachial and renal arteries in patients with hypertension, obesity, and renal disease [17]. Clinical trials are currently ongoing to examine the effects of the acute systemic infusion of Ang-(1-7) on blood pressure, sympathetic activity, and vasodilation in hypertensive, obese hypertensive, and aging populations (NCT06482853, NCT05301192). In terms of metabolic outcomes, a recent study showed that lower circulating Ang-(1-7) levels are associated with a higher body mass index, suggesting a connection between Ang-(1-7) deficiency and obesity [121]. Clinical trials are in progress to further examine this connection and determine the impact of acute Ang-(1-7) administration on metabolic outcomes, including energy balance and glucose homeostasis (e.g., NCT02646475, NCT03777215). Given the short half-life of the Ang-(1-7) peptide, novel therapies have also been developed for the more long-term targeting of Ang-(1-7) pathways (e.g., stable analogs, oral formulations, ACE2 activators, MasR agonists). These therapies are currently being tested in clinical trials, with initial reports showing improvement in cardiovascular and other outcomes in patient populations [122]. The metabolic effects of these novel therapies remain to be explored in clinical populations and should be a focus of future research, particularly given the positive integrated cardiometabolic effects of Ang-(1-7) observed in animal models. Whether Ang-(1-7) engages peripheral versus central mechanisms of action to improve cardiovascular and metabolic outcomes should also be explored, although this is more difficult to dissect in humans.

## 7. Conclusions and Future Directions for Research

Overall, emerging evidence shows that Ang-(1-7) is protective for both cardiovascular and metabolic outcomes in animal models, and thus may provide an alternate approach to target the RAS in obesity hypertension without eliciting the adverse side effects seen with traditional RAS inhibitors. The protective effects of Ang-(1-7) appear to involve a central mechanism of action, as MasR is widely expressed throughout the central nervous system, and icv infusion of this hormone lowers blood pressure, restores cardiac autonomic balance, and improves glucose homeostasis and adipose thermogenesis. When attempting to clarify the specific brain regions mediating these effects, the results are less clear, as Ang-(1-7) can either decrease or increase blood pressure depending on the specific brain region studied (Table 1). In some brainstem and hypothalamic regions, Ang-(1-7) elicits similar cardiovascular effects as Ang II, although via different cellular and neurotransmitter mechanisms, highlighting the complexity of these neural circuits. This occurs despite the known opposing cardiometabolic actions of these two peptides when administered systemically. These disparate findings could, in part, reflect the use of anesthesia and the high doses often used for acute microinjection studies as well as the lack of cell-type specificity in pharmacological targeting. Additional studies are needed to clarify the differences in outcomes between icv and brain-site-specific administration, as well as to identify the effects of Ang-(1-7) on additional brain regions and downstream targets, particularly those involved in parasympathetic regulation. While there have been several studies describing cardiovascular outcomes following central Ang-(1-7) administration, more research is needed to understand how these regions control metabolic outcomes, as well as the complex interactions between metabolic and blood pressure responses.

As described in Section 6, the translatability of these findings is currently being explored, with active clinical trials examining the cardiovascular and metabolic effects of acute systemic Ang-(1-7) infusion, as well as the cardiovascular actions of more stable Ang-(1-7) targeting therapies. Additional research is also needed to understand sex differences in systemic and central Ang-(1-7) cardiometabolic actions. While there are clear sex differences in the development, progression, and management of CVD [123], sex and gender have only recently been recognized as important variables to study in cardiovascular research [124]. Recent clinical studies have demonstrated sex-dependent associations between adiposity and cardiovascular risk, with the increased risk observed in males associated with enhanced activity of the vasoconstrictor arm of the RAS [125,126]. As recently reviewed by our group, despite having higher adiposity and obesity prevalence, females are often protected from the cardiovascular and metabolic complications of obesity [15,127]. The cardiovascular protection appears to result from multiple factors, including the presence of estrogen, reduced Ang II pathway activation, and enhanced Ang-(1-7) pathways. Despite this, there are few female comparison studies for metabolic outcomes, and the clinical data for sex differences in Ang-(1-7) pathways are lacking. Consistent with this, most of the studies cited in this review examining the central effects of Ang-(1-7) were only conducted in male animal models. Thus, additional research is critically needed to examine the actions of Ang-(1-7) in female animal models, including its actions on the central pathways involved in cardiometabolic regulation, as well as in women with obesity and related cardiovascular complications.

## Figures and Tables

**Table 1:** Summary of Ang-(1-7) route of administration and resulting cardiometabolic outcomes.

Location	Blood Pressure	Sympathetic Activity	BRS	Metabolic Outcomes
Systemic	⇓	⇓	⇑	⇑
Icv	⇓	⇓	⇑	⇑
Intra-NTS	⇓	UNK	⇑	UNK
Intra-CVLM	⇓	UNK	UNK	UNK
Intra-RVLM	⇑	⇑	UNK	UNK
Intra-ARC	UNK	UNK	UNK	UNK
Intra-PVN	⇑	⇑	UNK	UNK

Icv: intracerebroventricular; NTS: nucleus tractus solitarius; CVLM: caudal ventrolateral medulla; RVLM: rostral ventrolateral medulla; ARC: arcuate nucleus of hypothalamus; PVN: paraventricular nucleus of hypothalamus; BRS: baroreflex sensitivity; ⇑: increases; ⇓: decreases; UNK: unknown.

## Data Availability

No new data were created for this review article.

## References

[1] Esunge P.M. (1991). From Blood Pressure to Hypertension: The History of Research. J. R. Soc. Med..

[2] Saklayen M.G., Deshpande N.V. (2016). Timeline of History of Hypertension Treatment. Front. Cardiovasc. Med..

[3] Aird W.C. (2011). Discovery of the Cardiovascular System: From Galen to William Harvey. J. Thromb. Haemost..

[4] Jones D.S., Podolsky S.H., Greene J.A. (2012). The Burden of Disease and the Changing Task of Medicine. N. Engl. J. Med..

[5] Dalen J.E., Alpert J.S., Goldberg R.J., Weinstein R.S. (2014). The Epidemic of the 20th Century: Coronary Heart Disease. Am. J. Med..

[6] Mills K.T., Stefanescu A., He J. (2020). The Global Epidemiology of Hypertension. Nat. Rev. Nephrol..

[7] Whelton P.K., Carey R.M., Aronow W.S., Casey D.E., Collins K.J., Dennison Himmelfarb C., DePalma S.M., Gidding S., Jamerson K.A., Jones D.W. (2018). 2017 ACC/AHA/AAPA/ABC/ACPM/AGS/APhA/ASH/ASPC/NMA/PCNA Guideline for the Prevention, Detection, Evaluation, and Management of High Blood Pressure in Adults. J. Am. Coll. Cardiol..

[8] Parvanova A., Reseghetti E., Abbate M., Ruggenenti P. (2024). Mechanisms and Treatment of Obesity-Related Hypertension-Part 1: Mechanisms. Clin. Kidney J..

[9] Shariq O.A., McKenzie T.J. (2020). Obesity-Related Hypertension: A Review of Pathophysiology, Management, and the Role of Metabolic Surgery. Gland. Surg..

[10] Marketou M., Gupta Y., Jain S., Vardas P. (2017). Differential Metabolic Effects of Beta-Blockers: An Updated Systematic Review of Nebivolol. Curr. Hypertens. Rep..

[11] Cohen J.B., Gadde K.M. (2019). Weight Loss Medications in the Treatment of Obesity and Hypertension. Curr. Hypertens. Rep..

[12] Müller T.D., Blüher M., Tschöp M.H., DiMarchi R.D. (2022). Anti-Obesity Drug Discovery: Advances and Challenges. Nat. Rev. Drug Discov..

[13] Miller A.J., Arnold A.C. (2019). The Renin–Angiotensin System in Cardiovascular Autonomic Control: Recent Developments and Clinical Implications. Clin. Auton. Res..

[14] Dominici F.P., Gironacci M.M., Narvaez Pardo J.A. (2024). Therapeutic Opportunities in Targeting the Protective Arm of the Renin-Angiotensin System to Improve Insulin Sensitivity: A Mechanistic Review. Hypertens. Res..

[15] White M.C., Fleeman R., Arnold A.C. (2019). Sex Differences in the Metabolic Effects of the Renin-Angiotensin System. Biol. Sex. Differ..

[16] Santos R.A. (2014). Angiotensin-(1-7). Hypertension.

[17] Medina D., Arnold A.C. (2019). Angiotensin-(1-7): Translational Avenues in Cardiovascular Control. Am. J. Hypertens..

[18] Wu C.-H., Mohammadmoradi S., Chen J.Z., Sawada H., Daugherty A., Lu H.S. (2018). Renin-Angiotensin System and Cardiovascular Functions. Arterioscler. Thromb. Vasc. Biol..

[19] Favre G.A., Esnault V.L.M., Van Obberghen E. (2015). Modulation of Glucose Metabolism by the Renin-Angiotensin-Aldosterone System. Am. J. Physiol. Endocrinol. Metab..

[20] Swiderski J., Gadanec L.K., Apostolopoulos V., Moore G.J., Kelaidonis K., Matsoukas J.M., Zulli A. (2023). Role of Angiotensin II in Cardiovascular Diseases: Introducing Bisartans as a Novel Therapy for Coronavirus 2019. Biomolecules.

[21] Ramalingam L., Menikdiwela K., LeMieux M., Dufour J.M., Kaur G., Kalupahana N., Moustaid-Moussa N. (2017). The Renin Angiotensin System, Oxidative Stress and Mitochondrial Function in Obesity and Insulin Resistance. Biochim. Biophys. Acta Mol. Basis Dis..

[22] Parikh J.S., Randhawa A.K., Wharton S., Edgell H., Kuk J.L. (2018). The Association between Antihypertensive Medication Use and Blood Pressure is Influenced by Obesity. J. Obes..

[23] Acelajado M.C., Hughes Z.H., Oparil S., Calhoun D.A. (2019). Treatment of Resistant and Refractory Hypertension. Circ. Res..

[24] Arendse L.B., Danser A.H.J., Poglitsch M., Touyz R.M., Burnett J.C., Llorens-Cortes C., Ehlers M.R., Sturrock E.D. (2019). Novel Therapeutic Approaches Targeting the Renin-Angiotensin System and Associated Peptides in Hypertension and Heart Failure. Pharmacol. Rev..

[25] Pinto B., Jadhav U., Singhai P., Sadhanandham S., Shah N. (2020). ACEI-Induced Cough: A Review of Current Evidence and Its Practical Implications for Optimal CV Risk Reduction. Indian Heart J..

[26] Borghi C., Cicero A.F., Agnoletti D., Fiorini G. (2023). Pathophysiology of Cough with Angiotensin-Converting Enzyme Inhibitors: How to Explain within-Class Differences?. Eur. J. Intern. Med..

[27] Ostchega Y., Fryar C.D., Nwankwo T., Nguyen D.T. (2020). Hypertension Prevalence Among Adults Aged 18 and Over: United States, 2017–2018.

[28] Allred A.J., Diz D.I., Ferrario C.M., Chappell M.C. (2000). Pathways for Angiotensin-(1-7) Metabolism in Pulmonary and Renal Tissues. Am. J. Physiol. Ren. Physiol..

[29] Loloi J., Miller A.J., Bingaman S.S., Silberman Y., Arnold A.C. (2018). Angiotensin-(1-7) Contributes to Insulin-Sensitizing Effects of Angiotensin-Converting Enzyme Inhibition in Obese Mice. Am. J. Physiol. Endocrinol. Metab..

[30] Santos R.A.S., Sampaio W.O., Alzamora A.C., Motta-Santos D., Alenina N., Bader M., Campagnole-Santos M.J. (2018). The ACE2/Angiotensin-(1-7)/MAS Axis of the Renin-Angiotensin System: Focus on Angiotensin-(1-7). Physiol. Rev..

[31] Patel V.B., Zhong J.-C., Grant M.B., Oudit G.Y. (2016). Role of the ACE2/Angiotensin 1–7 Axis of the Renin–Angiotensin System in Heart Failure. Circ. Res..

[32] Regenhardt R.W., Desland F., Mecca A.P., Pioquinto D.J., Afzal A., Mocco J., Sumners C. (2013). Anti-Inflammatory Effects of Angiotensin-(1-7) in Ischemic Stroke. Neuropharmacology.

[33] Benter I.F., Ferrario C.M., Morris M., Diz D.I. (1995). Antihypertensive Actions of Angiotensin-(1-7) in Spontaneously Hypertensive Rats. Am. J. Physiol..

[34] Loot A.E., Roks A.J.M., Henning R.H., Tio R.A., Suurmeijer A.J.H., Boomsma F., van Gilst W.H. (2002). Angiotensin-(1-7) Attenuates the Development of Heart Failure after Myocardial Infarction in Rats. Circulation.

[35] Morimoto H., Mori J., Nakajima H., Kawabe Y., Tsuma Y., Fukuhara S., Kodo K., Ikoma K., Matoba S., Oudit G.Y. (2018). Angiotensin 1–7 Stimulates Brown Adipose Tissue and Reduces Diet-Induced Obesity. Am. J. Physiol. Endocrinol. Metab..

[36] Zhang F., Ren X., Zhao M., Zhou B., Han Y. (2016). Angiotensin-(1-7) Abrogates Angiotensin II-Induced Proliferation, Migration and Inflammation in VSMCs through Inactivation of ROS-Mediated PI3K/Akt and MAPK/ERK Signaling Pathways. Sci. Rep..

[37] Ni J., Yang F., Huang X., Meng J., Chen J., Bader M., Penninger J.M., Fung E., Yu X., Lan H. (2020). Dual Deficiency of Angiotensin-converting Enzyme-2 and Mas Receptor Enhances Angiotensin II-induced Hypertension and Hypertensive Nephropathy. J. Cell. Mol. Med..

[38] Metzger R., Bader M., Ludwig T., Berberich C., Bunnemann B., Ganten D. (1995). Expression of the Mouse and Rat Mas Proto-Oncogene in the Brain and Peripheral Tissues. FEBS Lett..

[39] Santos R.A.S., Simoes e Silva A.C., Maric C., Silva D.M.R., Machado R.P., de Buhr I., Heringer-Walther S., Pinheiro S.V.B., Lopes M.T., Bader M. (2003). Angiotensin-(1-7) Is an Endogenous Ligand for the G Protein-Coupled Receptor Mas. Proc. Natl. Acad. Sci. USA.

[40] Sampaio W.O., Henrique de Castro C., Santos R.A.S., Schiffrin E.L., Touyz R.M. (2007). Angiotensin-(1-7) Counterregulates Angiotensin II Signaling in Human Endothelial Cells. Hypertension.

[41] Sampaio W.O., Souza dos Santos R.A., Faria-Silva R., da Mata Machado L.T., Schiffrin E.L., Touyz R.M. (2007). Angiotensin-(1-7) through Receptor Mas Mediates Endothelial Nitric Oxide Synthase Activation via Akt-Dependent Pathways. Hypertension.

[42] McCollum L.T., Gallagher P.E., Ann Tallant E. (2012). Angiotensin-(1-7) Attenuates Angiotensin II-Induced Cardiac Remodeling Associated with Upregulation of Dual-Specificity Phosphatase 1. Am. J. Physiol. Heart Circ. Physiol..

[43] Povlsen A., Grimm D., Wehland M., Infanger M., Krüger M. (2020). The Vasoactive Mas Receptor in Essential Hypertension. J. Clin. Med..

[44] Bader M., Alenina N., Young D., Santos R.A.S., Touyz R.M. (2018). The Meaning of Mas. Hypertension.

[45] Galandrin S., Denis C., Boularan C., Marie J., M’Kadmi C., Pilette C., Dubroca C., Nicaise Y., Seguelas M.-H., N’Guyen D. (2016). Cardioprotective Angiotensin-(1–7) Peptide Acts as a Natural-Biased Ligand at the Angiotensin II Type 1 Receptor. Hypertension.

[46] Teixeira L.B., Parreiras-E-Silva L.T., Bruder-Nascimento T., Duarte D.A., Simões S.C., Costa R.M., Rodríguez D.Y., Ferreira P.A.B., Silva C.A.A., Abrao E.P. (2017). Ang-(1-7) Is an Endogenous β-Arrestin-Biased Agonist of the AT1 Receptor with Protective Action in Cardiac Hypertrophy. Sci. Rep..

[47] Paula R.D., Lima C.V., Khosla M.C., Santos R.A. (1995). Angiotensin-(1-7) Potentiates the Hypotensive Effect of Bradykinin in Conscious Rats. Hypertension.

[48] Walters P.E., Gaspari T.A., Widdop R.E. (2005). Angiotensin-(1-7) Acts as a Vasodepressor Agent via Angiotensin II Type 2 Receptors in Conscious Rats. Hypertension.

[49] Feterik K., Smith L., Katusic Z.S. (2000). Angiotensin-(1-7) Causes Endothelium-Dependent Relaxation in Canine Middle Cerebral Artery. Brain Res..

[50] Tetzner A., Gebolys K., Meinert C., Klein S., Uhlich A., Trebicka J., Villacañas Ó., Walther T. (2016). G-Protein-Coupled Receptor MrgD Is a Receptor for Angiotensin-(1-7) Involving Adenylyl Cyclase, cAMP, and Phosphokinase A. Hypertension.

[51] Bayorh M.A., Eatman D., Walton M., Socci R.R., Thierry-Palmer M., Emmett N. (2002). 1A-779 Attenuates Angiotensin-(1-7) Depressor Response in Salt-Induced Hypertensive Rats. Peptides.

[52] Liu C., Yang C.-X., Chen X.-R., Liu B.-X., Li Y., Wang X.-Z., Sun W., Li P., Kong X.-Q. (2018). Alamandine Attenuates Hypertension and Cardiac Hypertrophy in Hypertensive Rats. Amino Acids.

[53] Gong J., Luo M., Yong Y., Zhong S., Li P. (2022). Alamandine Alleviates Hypertension and Renal Damage via Oxidative-Stress Attenuation in Dahl Rats. Cell Death Discov..

[54] de Jesus I.C.G., Scalzo S., Alves F., Marques K., Rocha-Resende C., Bader M., Santos R.A.S., Guatimosim S. (2018). Alamandine Acts via MrgD to Induce AMPK/NO Activation against ANG II Hypertrophy in Cardiomyocytes. Am. J. Physiol. Cell Physiol..

[55] Williams I.M., Otero Y.F., Bracy D.P., Wasserman D.H., Biaggioni I., Arnold A.C. (2016). Chronic Angiotensin-(1-7) Improves Insulin Sensitivity in High-Fat Fed Mice Independent of Blood Pressure. Hypertension.

[56] Barbosa M.A., Barbosa C.M., Lima T.C., Dos Santos R.A.S., Alzamora A.C. (2020). The Novel Angiotensin-(1-7) Analog, A-1317, Improves Insulin Resistance by Restoring Pancreatic β-Cell Functionality in Rats with Metabolic Syndrome. Front. Pharmacol..

[57] Cerri G.C., Santos S.H.S., Bader M., Santos R.A.S. (2023). Brown Adipose Tissue Transcriptome Unveils an Important Role of the Beta-Alanine/Alamandine Receptor, MrgD, in Metabolism. J. Nutr. Biochem..

[58] Mecca A.P., Regenhardt R.W., O’Connor T.E., Joseph J.P., Raizada M.K., Katovich M.J., Sumners C. (2011). Cerebroprotection by Angiotensin-(1-7) in Endothelin-1-Induced Ischaemic Stroke. Exp. Physiol..

[59] Bruhns R.P., Sulaiman M.I., Gaub M., Bae E.H., Davidson Knapp R.B., Larson A.R., Smith A., Coleman D.L., Staatz W.D., Sandweiss A.J. (2022). Angiotensin-(1-7) Improves Cognitive Function and Reduces Inflammation in Mice Following Mild Traumatic Brain Injury. Front. Behav. Neurosci..

[60] Chittimalli K., Jahan J., Sakamuri A., McAdams Z.L., Ericsson A.C., Jarajapu Y.P.R. (2023). Restoration of the Gut Barrier Integrity and Restructuring of the Gut Microbiome in Aging by Angiotensin-(1-7). Clin. Sci..

[61] Schütten M.T.J., Houben A.J.H.M., de Leeuw P.W., Stehouwer C.D.A. (2017). The Link Between Adipose Tissue Renin-Angiotensin-Aldosterone System Signaling and Obesity-Associated Hypertension. Physiology.

[62] Becker L.K., Etelvino G.M., Walther T., Santos R.A.S., Campagnole-Santos M.J. (2007). Immunofluorescence Localization of the Receptor Mas in Cardiovascular-Related Areas of the Rat Brain. Am. J. Physiol. Heart Circ. Physiol..

[63] Fleegal-DeMotta M.A., Doghu S., Banks W.A. (2009). Angiotensin II Modulates BBB Permeability via Activation of the AT_1_ Receptor in Brain Endothelial Cells. J. Cereb. Blood Flow. Metab..

[64] Feng Z., Fang C., Ma Y., Chang J. (2024). Obesity-Induced Blood-Brain Barrier Dysfunction: Phenotypes and Mechanisms. J. Neuroinflamm..

[65] Biancardi V.C., Stern J.E. (2016). Compromised Blood-Brain Barrier Permeability: Novel Mechanism by Which Circulating Angiotensin II Signals to Sympathoexcitatory Centres during Hypertension. J. Physiol..

[66] Rhea E.M., Salameh T.S., Logsdon A.F., Hanson A.J., Erickson M.A., Banks W.A. (2017). Blood-Brain Barriers in Obesity. AAPS J..

[67] Salas-Venegas V., Flores-Torres R.P., Rodríguez-Cortés Y.M., Rodríguez-Retana D., Ramírez-Carreto R.J., Concepción-Carrillo L.E., Pérez-Flores L.J., Alarcón-Aguilar A., López-Díazguerrero N.E., Gómez-González B. (2022). The Obese Brain: Mechanisms of Systemic and Local Inflammation, and Interventions to Reverse the Cognitive Deficit. Front. Integr. Neurosci..

[68] Biancardi V.C., Son S.J., Ahmadi S., Filosa J.A., Stern J.E. (2014). Circulating Angiotensin II Gains Access to the Hypothalamus and Brain Stem during Hypertension via Breakdown of the Blood-Brain Barrier. Hypertension.

[69] Mendoza A., Lazartigues E. (2015). The Compensatory Renin-Angiotensin System in the Central Regulation of Arterial Pressure: New Avenues and New Challenges. Ther. Adv. Cardiovasc. Dis..

[70] Chappell M.C., Brosnihan K.B., Diz D.I., Ferrario C.M. (1989). Identification of Angiotensin-(1-7) in Rat Brain. J. Biol. Chem..

[71] Jackson L., Eldahshan W., Fagan S.C., Ergul A. (2018). Within the Brain: The Renin Angiotensin System. Int. J. Mol. Sci..

[72] Freund M., Walther T., von Bohlen und Halbach O. (2012). Immunohistochemical Localization of the Angiotensin-(1-7) Receptor Mas in the Murine Forebrain. Cell Tissue Res..

[73] Campagnole-Santos M.J., Heringer S.B., Batista E.N., Khosla M.C., Santos R.A. (1992). Differential Baroreceptor Reflex Modulation by Centrally Infused Angiotensin Peptides. Am. J. Physiol..

[74] Nautiyal M., Shaltout H.A., de Lima D.C., do Nascimento K., Chappell M.C., Diz D.I. (2012). Central Angiotensin-(1-7) Improves Vagal Function Independent of Blood Pressure in Hypertensive (mRen2)27 Rats. Hypertension.

[75] Mahon J.M., Allen M., Herbert J., Fitzsimons J.T. (1995). The Association of Thirst, Sodium Appetite and Vasopressin Release with c-Fos Expression in the Forebrain of the Rat after Intracerebroventricular Injection of Angiotensin II, Angiotensin-(1-7) or Carbachol. Neuroscience.

[76] Guimaraes P.S., Santiago N.M., Xavier C.H., Velloso E.P.P., Fontes M.A.P., Santos R.A.S., Campagnole-Santos M.J. (2012). Chronic Infusion of Angiotensin-(1-7) into the Lateral Ventricle of the Brain Attenuates Hypertension in DOCA-Salt Rats. Am. J. Physiol. Heart Circ. Physiol..

[77] Kangussu L.M., Guimaraes P.S., Nadu A.P., Melo M.B., Santos R.A.S., Campagnole-Santos M.J. (2015). Activation of Angiotensin-(1-7)/Mas Axis in the Brain Lowers Blood Pressure and Attenuates Cardiac Remodeling in Hypertensive Transgenic (mRen2)27 Rats. Neuropharmacology.

[78] Jiang T., Gao L., Shi J., Lu J., Wang Y., Zhang Y. (2013). Angiotensin-(1-7) Modulates Renin-Angiotensin System Associated with Reducing Oxidative Stress and Attenuating Neuronal Apoptosis in the Brain of Hypertensive Rats. Pharmacol. Res..

[79] Kangussu L.M., Melo-Braga M.N., de Souza Lima B.S., Santos R.A.S., de Andrade H.M., Campagnole-Santos M.J. (2021). Angiotensin-(1-7) Central Mechanisms After ICV Infusion in Hypertensive Transgenic (mRen2)27 Rats. Front. Neurosci..

[80] Hendricks A.S., Lawson M.J., Figueroa J.P., Chappell M.C., Diz D.I., Shaltout H.A. (2019). Central ANG-(1-7) Infusion Improves Blood Pressure Regulation in Antenatal Betamethasone-Exposed Sheep and Reveals Sex-Dependent Effects on Oxidative Stress. Am. J. Physiol. Heart Circ. Physiol..

[81] Villela D.C., Passos-Silva D.G., Santos R.A.S. (2014). Alamandine: A New Member of the Angiotensin Family. Curr. Opin. Nephrol. Hypertens..

[82] Guimaraes P.S., Oliveira M.F., Braga J.F., Nadu A.P., Schreihofer A., Santos R.A.S., Campagnole-Santos M.J. (2014). Increasing Angiotensin-(1-7) Levels in the Brain Attenuates Metabolic Syndrome-Related Risks in Fructose-Fed Rats. Hypertension.

[83] Evangelista F.S., Bartness T.J. (2023). Central Angiotensin 1-7 Triggers Brown Fat Thermogenesis. Physiol. Rep..

[84] Sapouckey S.A., Deng G., Sigmund C.D., Grobe J.L. (2017). Potential Mechanisms of Hypothalamic Renin-Angiotensin System Activation by Leptin and DOCA-Salt for the Control of Resting Metabolism. Physiol. Genom..

[85] Dampney R.A., Michelini L.C., Li D.-P., Pan H.-L. (2018). Regulation of Sympathetic Vasomotor Activity by the Hypothalamic Paraventricular Nucleus in Normotensive and Hypertensive States. Am. J. Physiol.-Heart Circ. Physiol..

[86] Dampney R.A.L. (2016). Central Neural Control of the Cardiovascular System: Current Perspectives. Adv. Physiol. Educ..

[87] Zoccal D.B., Furuya W.I., Bassi M., Colombari D.S.A., Colombari E. (2014). The Nucleus of the Solitary Tract and the Coordination of Respiratory and Sympathetic Activities. Front. Physiol..

[88] Campagnole-Santos M.J., Diz D.I., Santos R.A., Khosla M.C., Brosnihan K.B., Ferrario C.M. (1989). Cardiovascular Effects of Angiotensin-(1-7) Injected into the Dorsal Medulla of Rats. Am. J. Physiol..

[89] Diz D.I., Garcia-Espinosa M.A., Gallagher P.E., Ganten D., Ferrario C.M., Averill D.B. (2008). Angiotensin-(1-7) and Baroreflex Function in Nucleus Tractus Solitarii of (mRen2)27 Transgenic Rats. J. Cardiovasc. Pharmacol..

[90] Chaves G.Z., Caligiorne S.M., Santos R.A., Khosla M.C., Campagnole-Santos M.J. (2000). Modulation of the Baroreflex Control of Heart Rate by Angiotensin-(1-7) at the Nucleus Tractus Solitarii of Normotensive and Spontaneously Hypertensive Rats. J. Hypertens..

[91] Moreira T.S., Sato M.A., Takakura A.C.T., Menani J.V., Colombari E. (2005). Role of Pressor Mechanisms from the NTS and CVLM in Control of Arterial Pressure. Am. J. Physiol. Regul. Integr. Comp. Physiol..

[92] Cravo S.L., Morrison S.F., Reis D.J. (1991). Differentiation of Two Cardiovascular Regions within Caudal Ventrolateral Medulla. Am. J. Physiol..

[93] Cangussu L.M., de Castro U.G.M., do Pilar Machado R., Silva M.E., Ferreira P.M., dos Santos R.A.S., Campagnole-Santos M.J., Alzamora A.C. (2009). Angiotensin-(1-7) Antagonist, A-779, Microinjection into the Caudal Ventrolateral Medulla of Renovascular Hypertensive Rats Restores Baroreflex Bradycardia. Peptides.

[94] de Sousa G.G., Barbosa M.A., Barbosa C.M., Lima T.C., Souza Dos Santos R.A., Campagnole-Santos M.J., Alzamora A.C. (2020). Different Reactive Species Modulate the Hypotensive Effect Triggered by Angiotensins at CVLM of 2K1C Hypertensive Rats. Peptides.

[95] Cerrato B.D., Frasch A.P., Nakagawa P., Longo-Carbajosa N., Peña C., Höcht C., Gironacci M.M. (2012). Angiotensin-(1-7) Upregulates Central Nitric Oxide Synthase in Spontaneously Hypertensive Rats. Brain Res..

[96] Alzamora A.C., Santos R.a.S., Campagnole-Santos M.J. (2002). Hypotensive Effect of ANG II and ANG-(1-7) at the Caudal Ventrolateral Medulla Involves Different Mechanisms. Am. J. Physiol. Regul. Integr. Comp. Physiol..

[97] Soares E.R., Barbosa C.M., Campagnole-Santos M.J., Santos R.a.S., Alzamora A.C. (2017). Hypotensive Effect Induced by Microinjection of Alamandine, a Derivative of Angiotensin-(1-7), into Caudal Ventrolateral Medulla of 2K1C Hypertensive Rats. Peptides.

[98] Guyenet P.G., Stornetta R.L., Holloway B.B., Souza G.M.P.R., Abbott S.B.G. (2018). Rostral Ventrolateral Medulla and Hypertension. Hypertension.

[99] Du D., Chen J., Liu M., Zhu M., Jing H., Fang J., Shen L., Zhu D., Yu J., Wang J. (2013). The Effects of Angiotensin II and Angiotensin-(1-7) in the Rostral Ventrolateral Medulla of Rats on Stress-Induced Hypertension. PLoS ONE.

[100] Lautner R.Q., Villela D.C., Fraga-Silva R.A., Silva N., Verano-Braga T., Costa-Fraga F., Jankowski J., Jankowski V., Sousa F., Alzamora A. (2013). Discovery and Characterization of Alamandine: A Novel Component of the Renin–Angiotensin System. Circ. Res..

[101] Bilodeau M.S., Leiter J.C. (2018). Angiotensin 1-7 in the Rostro-Ventrolateral Medulla Increases Blood Pressure and Splanchnic Sympathetic Nerve Activity in Anesthetized Rats. Respir. Physiol. Neurobiol..

[102] Li P., Sun H.-J., Cui B.-P., Zhou Y.-B., Han Y. (2013). Angiotensin-(1-7) in the Rostral Ventrolateral Medulla Modulates Enhanced Cardiac Sympathetic Afferent Reflex and Sympathetic Activation in Renovascular Hypertensive Rats. Hypertension.

[103] Zhou L.-M., Shi Z., Gao J., Han Y., Yuan N., Gao X.-Y., Zhu G.-Q. (2010). Angiotensin-(1-7) and Angiotensin II in the Rostral Ventrolateral Medulla Modulate the Cardiac Sympathetic Afferent Reflex and Sympathetic Activity in Rats. Pflug. Arch..

[104] Silva L.C., Fontes M.A., Campagnole-Santos M.J., Khosla M.C., Campos R.R., Guertzenstein P.G., Santos R.A. (1993). Cardiovascular Effects Produced by Micro-Injection of Angiotensin-(1-7) on Vasopressor and Vasodepressor Sites of the Ventrolateral Medulla. Brain Res..

[105] Yamazato M., Yamazato Y., Sun C., Diez-Freire C., Raizada M.K. (2007). Overexpression of Angiotensin-Converting Enzyme 2 in the Rostral Ventrolateral Medulla Causes Long-Term Decrease in Blood Pressure in the Spontaneously Hypertensive Rats. Hypertension.

[106] Rahmouni K. (2016). Cardiovascular Regulation by the Arcuate Nucleus of the Hypothalamus: Neurocircuitry and Signaling Systems. Hypertension.

[107] Deng Y., Deng G., Grobe J.L., Cui H. (2021). Hypothalamic GPCR Signaling Pathways in Cardiometabolic Control. Front. Physiol..

[108] Mehay D., Silberman Y., Arnold A.C. (2021). The Arcuate Nucleus of the Hypothalamus and Metabolic Regulation: An Emerging Role for Renin–Angiotensin Pathways. Int. J. Mol. Sci..

[109] Lawton S.B.R., Wagner V.A., Nakagawa P., Segar J.L., Sigmund C.D., Morselli L.L., Grobe J.L. (2024). Angiotensin in the Arcuate: Mechanisms Integrating Cardiometabolic Control: The 2022 COH Mid-Career Award for Research Excellence. Hypertension.

[110] Shi Z., Stornetta D.S., Stornetta R.L., Brooks V.L. (2022). Arcuate Angiotensin II Increases Arterial Pressure via Coordinated Increases in Sympathetic Nerve Activity and Vasopressin Secretion. eNeuro.

[111] Savić B., Murphy D., Japundžić-Žigon N. (2022). The Paraventricular Nucleus of the Hypothalamus in Control of Blood Pressure and Blood Pressure Variability. Front. Physiol..

[112] Carmichael C.Y., Wainford R.D. (2015). Hypothalamic Signaling Mechanisms in Hypertension. Curr. Hypertens. Rep..

[113] Sun H.-J., Li P., Chen W.-W., Xiong X.-Q., Han Y. (2012). Angiotensin II and Angiotensin-(1-7) in Paraventricular Nucleus Modulate Cardiac Sympathetic Afferent Reflex in Renovascular Hypertensive Rats. PLoS ONE.

[114] Ren X., Zhang F., Zhao M., Zhao Z., Sun S., Fraidenburg D.R., Tang H., Han Y. (2017). Angiotensin-(1-7) in Paraventricular Nucleus Contributes to the Enhanced Cardiac Sympathetic Afferent Reflex and Sympathetic Activity in Chronic Heart Failure Rats. Cell Physiol. Biochem..

[115] Yu X.-J., Miao Y.-W., Li H.-B., Su Q., Liu K.-L., Fu L.-Y., Hou Y.-K., Shi X.-L., Li Y., Mu J.-J. (2019). Blockade of Endogenous Angiotensin-(1-7) in Hypothalamic Paraventricular Nucleus Attenuates High Salt-Induced Sympathoexcitation and Hypertension. Neurosci. Bull..

[116] Han Y., Sun H.-J., Li P., Gao Q., Zhou Y., Zhang F., Gao X.-Y., Zhu G.-Q. (2012). Angiotensin-(1-7) in Paraventricular Nucleus Modulates Sympathetic Activity and Cardiac Sympathetic Afferent Reflex in Renovascular Hypertensive Rats. PLoS ONE.

[117] Sriramula S., Cardinale J.P., Lazartigues E., Francis J. (2011). ACE2 Overexpression in the Paraventricular Nucleus Attenuates Angiotensin II-Induced Hypertension. Cardiovasc. Res..

[118] Shen Y.-H., Chen X.-R., Yang C.-X., Liu B.-X., Li P. (2018). Alamandine Injected into the Paraventricular Nucleus Increases Blood Pressure and Sympathetic Activation in Spontaneously Hypertensive Rats. Peptides.

[119] Nakagawa P., Gomez J., Grobe J.L., Sigmund C.D. (2020). The Renin-Angiotensin System in the Central Nervous System and Its Role in Blood Pressure Regulation. Curr. Hypertens. Rep..

[120] Durand M.J., Zinkevich N.S., Riedel M., Gutterman D.D., Nasci V.L., Salato V.K., Hijjawi J.B., Reuben C.F., North P.E., Beyer A.M. (2016). Vascular Actions of Angiotensin 1–7 in the Human Microcirculation: Novel Role for Telomerase. Arterioscler. Thromb. Vasc. Biol..

[121] Vargas-Castillo A., Tobon-Cornejo S., Del Valle-Mondragon L., Torre-Villalvazo I., Schcolnik-Cabrera A., Guevara-Cruz M., Pichardo-Ontiveros E., Fuentes-Romero R., Bader M., Alenina N. (2020). Angiotensin-(1-7) Induces Beige Fat Thermogenesis through the Mas Receptor. Metabolism.

[122] Bader M., Steckelings U.M., Alenina N., Santos R.A.S., Ferrario C.M. (2024). Alternative Renin-Angiotensin System. Hypertension.

[123] Regitz-Zagrosek V., Kararigas G. (2017). Mechanistic Pathways of Sex Differences in Cardiovascular Disease. Physiol. Rev..

[124] Usselman C.W., Lindsey M.L., Robinson A.T., Habecker B.A., Taylor C.E., Merryman W.D., Kimmerly D., Bender J.R., Regensteiner J.G., Moreau K.L. (2024). Guidelines on the Use of Sex and Gender in Cardiovascular Research. Am. J. Physiol. Heart Circ. Physiol..

[125] Kalenga C.Z., Ramesh S., Dumanski S.M., MacRae J.M., Nerenberg K., Metcalfe A., Sola D.Y., Ahmed S.B. (2022). Sex Influences the Effect of Adiposity on Arterial Stiffness and Renin-Angiotensin Aldosterone System Activity in Young Adults. Endocrinol. Diabetes Metab..

[126] Hundemer G.L., Agharazii M., Madore F., Piché M.-E., Gagnon C., Bussières A., St-Jean M., Leung A.A., Kline G.A., Sood M.M. (2024). Sex-Specific Associations of Aldosterone and Renin with Body Composition: A Population-Based Cohort Study. J. Clin. Endocrinol. Metab..

[127] Medina D., Mehay D., Arnold A.C. (2020). Sex Differences in Cardiovascular Actions of the Renin-Angiotensin System. Clin. Auton. Res..

